# Stochastic model highlights the impact of crystallinity on saccharification dynamics depending on plant chemotype and pre-treatment

**DOI:** 10.1371/journal.pone.0322367

**Published:** 2025-12-05

**Authors:** Partho Sakha De, Philipp M Grande, Henrike Heise, Holger Klose, Adélaïde Raguin

**Affiliations:** 1 Institute for Computational Cell Biology, Heinrich Heine University, Düsseldorf, Germany; 2 Bioeconomy Science Center (BioSC), c/o Forschungszentrum Jülich, Jülich, Germany; 3 Cluster of Excellence on Plant Sciences (CEPLAS), Heinrich Heine University, Düsseldorf, Germany; 4 Institute of Bio- and Geosciences (IBG) Plant Sciences (IBG-2), Forschungszentrum Jülich GmbH, Jülich, Germany; 5 Institute for Physical Biology, Heinrich Heine University, Düsseldorf, Germany; 6 Institute of Structural Biochemistry (IBI-7), Forschungszentrum Jülich GmbH, Jülich, Germany; 7 RWTH Aachen University, Aachen, Germany; 8 Laboratoire Bordelais de Recherche en Informatique (LaBRI - UMR 5800), CNRS, Univ. Bordeaux, Bordeaux INP, Talence, France; University of Sao Paulo, BRAZIL

## Abstract

Enzymatic saccharification of plant-sourced lignocellulosic biomass is a key step in biorefinery approaches. However, these biomasses in their raw form are quite recalcitrant, which invokes the need for pre-treatment processes aimed at not only increasing glucose conversion, but also better valorising non-carbohydrate biopolymers, such as lignin. Here, we use a two-fold computational and experimental approach to investigate enzymatic saccharification time-courses for three cellulosic substrates (i.e. AVICEL, a mixture of AVICEL with Organosolv lignin, and Sigmacell), and four plant-sourced lignocellulosic biomasses following three different conditions for each of them (i.e. untreated, OrganoCat pre-treated with a swelling step, and OrganoCat pre-treated without a swelling step), making a total of fifteen samples. Considering the specific composition of each substrate, the model successfully reproduces the saccharification dynamics for each of the fifteen samples. It additionally provides values for the parameter Crystallinity Fraction that faithfully replicate the substrate Crystallinity Indices experimentally determined by ssNMR. Importantly, we show that the Crystallinity Index of distinct biomasses is differently impacted by swelling, while the sugar release is consistently impacted by pre-treatment across biomasses. Eventually, both artificial cellulosic and plant-sourced lignocellulosic biomasses demonstrate that the sugar release is the result of the combination of the Crystallinity Fraction (the model parameter for experimentally measured ssNMR Crystallinity Index) and the digestibility ratio, the model parameter that represents in a coarse-grained manner complex spatial and structural features. Overall, our results stress the need for further experimental investigations that physically explain variations in the digestibility of crystalline bonds across biomasses and pre-treatment conditions. Additionally, we supplemented our work with theoretical investigations on a generic lignocellulosic substrate to highlight the roles of various model parameters in a qualitative manner.

## 1 Introduction

Lignocellulosic biomass is an abundant source of raw material for the production of biofuels and other valuable chemicals. In biorefinery processes, it is broken down by enzymatic saccharification to release the constituent simple monomeric sugars from the biopolymers. However, its recalcitrance to enzymatic saccharification is a major challenge that has been speculated to be impacted by various factors. Those range from the crystallinity of the substrate, to the inhibitory effect of lignin on the enzymes. For reducing recalcitrance, and hence enhancing saccharification yield, this biomass is subjected to chemical and physical pre-treatments, such as: acidic treatments involving phosphoric, oxalic, acetic, or sulphuric acids [1,2]; alkaline treatments involving slaked lime or ammonia fiber explosion/expansion (AFEX) [3–6], and exposure to steam or liquid hot water [7,8]. Alternative methods for instance rely on innovative enzymatic processes, e.g. the laccase system mediated by 1-hydroxybenzotriazole [9]. In this study, we specifically employ an Organosolv-like OrganoCat process, which involves a diluted acid, e.g. oxalic acid, 2,5-furandicarboxylic acid, or phosphoric acid to hydrolyse hemicelluloses, and *in situ* extract lignin into a second phase of 2-methyltetrahydrofuran (2-MTHF) [10–12]. In addition to experimental approaches, several models have been developed, at multiple scales, to understand the dynamics of enzymatic saccharification and decipher the effect of various structural and compositional properties of lignocellulose on its recalcitrance. Each of these approaches possesses its own set of advantages and disadvantages, which have been nicely summarised in the comprehensive review by Ciesielski et al. [13]. At the smallest scale, density functional theory (DFT), quantum mechanics/molecular mechanics (QM/MM), and molecular dynamics (MD) are typical methods [14–17]. They are used to address problems such as pyrolysis [18,19], the detailed structure and properties of lignocellulosic biomass [20], enzyme mechanisms [21,22], and the effects of lignin binding on cellulose and cellulase enzymes [17]. However, they are very demanding in computational resources, and for instance are unable to depict biopolymers at the scale of seconds. To counter these disadvantages, alternative approaches based on coarse-grained molecular dynamics have been employed with beads or pseudo atoms as elementary units [23]. Instead, considering glucose molecules as elementary units, Kumar and Murthy implemented Monte Carlo simulations to model the digestion of a cellulose bundle under the action of endoglucanase (EG), cellobiohydrolase (CBH), and *β*-glucosidase (BGL) [24,25]. They considered a substrate of crystalline cellulose with hemicellulose and lignin, but did not study the impact of crystallinity on the saccharification dynamics, and their simulation results displayed significant discrepancies with experimental data. Using Ordinary Differential Equations, Griggs et al. [26,27] developed a mechanistic and kinetic model to simulate the action of a cocktail of cellulase enzymes on a purely cellulosic substrate, while this framework can also be applied at a much higher (i.e. reactor) scale [28]. Their results showed the enzyme synergism, a good agreement with experimental cellulose chain length distributions from literature, and a semi-quantitative agreement with experimental saccharification time-courses. Notably, their model nonetheless neglected all constituents other than cellulose. With agent-based models, Vetharaniam et al. [29] and Asztalos et al. [30] both investigated enzymatic synergism, while respectively considering: (i) hemicellulosic sugars and crystalline cellulose; and (ii) inter-chain hydrogen bond breaking, hydrolysis of glycosidic bonds, and adsorption and desorption of the cellulases on the substrate. Yet, these studies oversimplify the substrate when neglecting the essential constituent lignin, and considering a two-dimensional and purely cellulosic substrate. To fill these gaps, and build a comprehensive model of lignocellulose and its enzymatic saccharification dynamics, we previously introduced a spatially resolved stochastic biophysical model of a lignocellulose microfibril [31], which has later on been made available as an advanced free, open-source, and user-friendly Web Application https://predig.cs.hhu.de/ [32]. The model accounted for the detailed biomass structure, the specific action of the enzymes, the crystallinity of cellulose and hemicellulose [33,34], and the role of lignin; and demonstrated an exceptional ability in quantitatively reproducing experimental saccharification time-courses.

Despite our previous work and all the advancements in the investigation of enzymatic degradation of lignocellulosic biomass, it remains unclear how crystallinity in distinct biomasses differently impacts on saccharification recalcitrance, and to which extent experimental measurements of crystallinity can explain saccharification dynamics. To answer these questions, in this study, we develop a two-fold (computational and experimental) approach and focus on the extraction of glucose from various biomasses, with no pre-treatment and two distinct pre-treatment conditions. Our findings suggest that the enzyme kinetics is strongly affected by not only the type of substrate i.e. pure cellulose *vs* lignocellulose, but also the pre-treatment each biomass is subjected to. Moreover, the model shows that even directly accounting for ssNMR measurements of the crystallinity index is insufficient to accurately reproduce the saccharification dynamics, which is however well captured when additionally considering in the model that crystalline bonds in different biomasses have different propensities to be digested, which impact on recalcitrance in a non-trivial way. Overall, we demonstrate the importance of modelling for rationalising experimental data relating to the structure and dynamics of plant biomass, a highly complex composite material.

## 2 Materials and methods

### 2.1 Extraction and analysis of lignocellulose

All chemicals were purchased from Carl Roth and Sigma-Aldrich (Germany) and used without further purification. The plant biomasses were the same set as published earlier [1]. The extraction and analysis procedures were carried out with slight modifications as outlined in our previous study [35]. In summary, the biomasses were reduced in size before subsequent treatment by grinding to a fine powder using a ball mill M 400 (Retsch, Haan, Germany) in a 50 mL metal beaker (30 s^−1^, 2 min). For each plant, 1 g of powder was extracted in 50 mL reaction tubes, and the resulting pellet was collected by centrifugation at 3,234 g for 5 minutes. The alcohol-insoluble residue (AIR) was utilised to quantify the content of crystalline cellulose and lignin. Lignin was determined as acetyl bromide soluble lignin (ABSL) and crystalline cellulose content was measured by the Updegraff method, like described by Foster et al. [36,37]. De-starched AIR (d-AIR) was employed to assess the content of matrix polysaccharides (MPS) through High-Performance Anion-Exchange Chromatography with Pulsed Amperometric Detection (HPAEC-PAD) analysis [35]. The composition of each of the samples is summarised in Table 1.

**Table 1:** Composition of cellulosic and lignocellulosic biomasses in various conditions: untreated, OrganoCat treated without swelling step (NS+OCAT), and OrganoCat treated with swelling step (YS+OCAT).

Biomass source	Pre-treatment	cellulose [%]	lignin [%]	MPS [%]	acetate [%]	total [%]
AVICEL	NA	80.6±0.78	11.3±0.75*	3.7±0.17	3.9±0.09	99.5
Sigmacell	NA	82.8±0.82	9.2±0.68*	4.0±0.18	3.6±0.13	99.2
Beech	untreated	45.3±1.7	24.7±2.0	22.8±3.5	5.1±0.5	97.9
	NS+OCAT	71.0±1.5	21.8±1.0	4.2±0.5	0.0±0.1	97.1
	YS+OCAT	68.6±1.5	23.8±2.5	1.9±0.1	0.0±0.0	94.3
Miscanthus	untreated	46.5±4.3	24.4±2.2	24.3±3.6	2.7±0.1	97.8
	NS+OCAT	71.9±2.0	20.9±1.1	5.9±0.8	0.0±0.0	98.7
	YS+OCAT	80.8±2.7	19.7±1.1	1.8±0.1	0.1±0.1	102.4
Sida (Sida hermaphrodita)	untreated	44.7±2.3	25.2±1.2	17.6±2.5	5.1±0.2	92.6
	NS+OCAT	62.1±0.9	18.2±1.5	6.6±0.7	0.1±0.2	87.1
	YS+OCAT	68.5±0.8	23.7±0.5	3.4±0.5	0.4±0.1	96.0
Walnut	untreated	31.4±4.0	28.4±1.9	15.7±3.2	4.6±0.2	80.2
	NS+OCAT	50.9±1.8	28.4±2.5	6.8±0.1	0.2±0.1	86.3
	YS+OCAT	50.0±1.0	29.3±0.3	3.5±0.6	0.2±0.1	83.0

**Note:**  Positive lignin results in AVICEL and Sigmacel are artifacts of the measurement and should not be considered.

### 2.2 Lignocellulose fractionation by OrganoCat pulping

*Without swelling.* In a high-pressure reactor with a volume of 20 mL, 500 mg of lignocellulose biomass, 5 mL of phosphoric acid (0.74 M) and 5 mL of 2-MTHF were introduced. To prevent 2-MTHF evaporation, the stainless-steel high-pressure reactor was sealed and pressurised with 10 bar of argon. The mixture was stirred for 3 hours at 140°C. After cooling the reactor to room temperature, the liquid phases were separated through decantation and the cellulose-enriched solid pulp was filtered. Sugar concentrations were determined using HPAEC-PAD. The solid residue was washed with distilled water until it reached a neutral pH and then dried until a constant weight was achieved. Lignin was obtained by evaporating the 2-MTHF and quantified through gravimetric analysis.

*With swelling.* In a high-pressure reactor with a volume of 20 mL, 500 mg of lignocellulose biomass, 250 μ L of ultra-pure water, and 250 μ L of phosphoric acid (85 wt%) were combined. This mixture was then heated to 80^°^C for 1 hour. Subsequently, 4.5 mL of ultra-pure water and 5 mL of 2-MTHF were introduced. To prevent 2-MTHF evaporation, the stainless-steel high-pressure reactor was sealed and pressurised with 10 bars of argon. The mixture was stirred for 3 hours at 140°C. After cooling the reactor to room temperature, the liquid phases were separated through decantation and the cellulose-enriched solid pulp was filtered. Sugar concentrations in the aqueous phase were determined using HPAEC-PAD. The solid residue was washed with distilled water until it reached a neutral pH and then dried until a constant weight was achieved. Lignin was obtained by evaporating the 2-MTHF and quantified through gravimetric analysis.

The OrganoCat treatment applied to the lignocelluloses did not result in instantaneous expansion and does not have a significant effect on the cellulose fibres, unlike for instance steam explosion, where intense heating leads to the explosive expansion of vapour, causing cell damage.

### 2.3 Enzymatic hydrolysis of cellulose pulp

Enzymatic hydrolysis and its subsequent analysis were performed following the protocols detailed in our previous study [38]. In brief, each of the cellulosic or lignocellulosic biomasses was suspended at a concentration of 20 g/L in a 0.1 M sodium citrate buffer with a pH of 4.5 (total volume of 1 mL). This suspension was maintained at a temperature of 50^°^C for specified amount of time. To initiate the enzymatic reaction, Accellerase^®^-1500 (containing 60 FPU/mL and 82 CBU/mL, Genencor, The Netherlands) was added at a volume of 1 vol%, relative to the buffer. Samples (0.3 mL) were withdrawn from the reactions at indicated times, heated to 100^°^C for 5 minutes to halt the enzymatic process, and subsequently stored at $-20^{\circ }$C until the colourimetric analysis was carried out. Sugar release was determined by employing the PAHBAH (4-hydroxybenzoic acid hydrazide) method, following the procedure outlined by Lever et al. [39]. The resulting reaction mixtures were diluted as needed to be in the range of the calibration curve. The absorbance was measured at 410 nm using a BioTek Power Wave HT UV/Vis spectrometer. All experiments were performed as single trials or with replicates, as specified in the text.

### 2.4 Crystallinity measurement via ssNMR

For solid-state NMR measurements, ca. 30 mg of the sample were packed into Bruker magic-angle spinning (MAS) rotors with an outer diameter of 3.2 mm. ^13^C Cross-Polarisation (CP) MAS spectra were recorded on a 14.1 T (600 MHz ${\;}^{1}\text{H}$ frequency) Bruker Avance wide bore spectrometer equipped with a 3.2 mm MAS triple resonance ${\;}^{1}\text{H}$, ${\;}^{13}\text{C}$, probe and a 14.1 T (600 MHz ${\;}^{1}\text{H}$ frequency) Bruker AVANCE NEO spectrometer equipped with a triple resonance HCN 3.2 mm MAS Efree probe. The CP contact time was 500 *μ*s, the MAS spinning speed was 11 kHz. Spinal-64 ${\;}^{1}\text{H}$ decoupling (RF field of 85 kHz) was applied during acquisition. Spectra were referenced externally to Sodium trimethylsilylpropanesulfonate (DSS) using adamantane as a secondary standard (the low frequency peak was set to 31.4 ppm).

### 2.5 Stochastic simulation model

**Fig 1:**
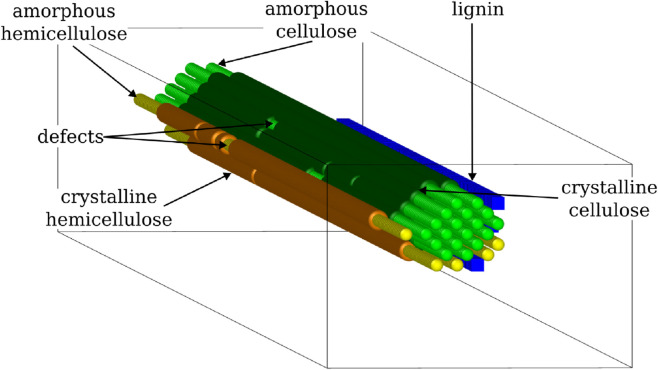
Perspective view of the modelled lignocellulose microfibril, with lignin in blue, hemicellulose in yellow, and cellulose in green. Both hemicellulose and cellulose can be either crystalline (dark colour) or amorphous (light colour), with amorphous regions typically located at the boundaries of the microfibril, or embedded in the crystalline region as ‘defect’ patches. These defects may for instance arise as a result of pre-treatments.

Our theoretical model [31] employs a Gillespie algorithm (see Sect 2.6 for details) to stochastically simulate the enzymatic saccharification process of a lignocellulose microfibril. It represents *in silico* the distinct bio-polymers (namely cellulose, hemicellulose, and lignin) constituting the biomass, as well as the three-dimensional configuration of the substrate. The physical structure of the substrate is depicted as a hexagonal-shaped core bundle of cellulose polymer chains of length 200 bonds, surrounded by two layers of randomly positioned hemicellulose and lignin polymers, as seen in Fig 1. The number of cellulose chains that form the core of a microfibril has been strongly debated for decades, and it is widely accepted that they form in multiples of six, i.e. 36, 24, or 18, with a recent preference for the latter one [40,41]. Since this number is known to vary for different species and developmental stages [41–43], and that the conclusions from the model we present here are not impaired by these considerations, we choose to simulate the system for eighteen cellulose chains. The inner polymer bonds are shielded from enzymatic action by the outer chains, until those get digested, giving physical access to the enzymes. Besides, the lignin and hemicellulose layers do not completely surround the cellulose core, but possess some gaps, as seen in Fig 1.

Biomass crystallinity is also known to have a strong impact on enzymatic saccharification. It is well-established that cellulose possesses a crystalline arrangement that negatively impacts saccharification. Even though there is no direct evidence of hemicellulose crystallinity, xylans have been known to partially bind to cellulose microfibrils, adopting a semi-crystalline arrangement [33]. It has also been suggested that hemicellulose adsorption to cellulose interferes with the saccharification process [34]. Furthermore, the hemicelluloses of the biomasses considered in our study are predominantly constituted of xylose. Thus, in our model, we consider both crystalline and amorphous regions for both cellulose and hemicellulose, so that the crystalline bonds are more difficult to enzymatically digest as compared to their amorphous counterparts. Crystallinity *versus* amorphousness is quantified using two distinct parameters: (i) the Crystallinity Fraction (noted CF) quantifies the number of crystalline bonds over the total number of bonds of a certain type (i.e. cellulose or hemicellulose), and (ii) the digestibility ratio (noted $r_{c,a}$) describes how much harder it is to digest a crystalline bond as compared to its amorphous counterpart, for a certain bond type (i.e. cellulose or hemicellulose). For instance, when $r_{c,a}=0$, it implies that crystalline bonds are not digestible at all, whereas when $r_{c,a}=1$, crystalline bonds are digested at the same rate as amorphous ones. We assume that the crystallinity of hemicellulose is induced by its direct vicinity to the highly ordered and crystalline cellulose chains. We also account for the non-productive adsorption of enzymes on lignin, independently of their specific type [44–47].

The microfibril is subjected to digestion by an enzyme cocktail consisting of endoglucanases (EGs), cellobiohydrolases (CBHs), *β*-glucosidases (BGLs), and hemicellulases (HCs). Endoglucanases can digest any exposed bond in the bulk of a cellulose chain, except the two endmost ones, at each end of the polymer chain [48]. Cellobiohydrolases attach to glucan chains’ ends from which they processively cleave-off cellobiose units [49–51]. *β*-glucosidases complete the saccharification process by splitting cellobiose into two glucose monomers [52,53]. In our model, we account for the cellulases’ specific mode of action, unlike for the hemicellulases that are considered as non-specific for the sake of simplicity, and capable of digesting any exposed digestible hemicellulosic bond. Our model also accounts for end product inhibition of the cellulases, such that free glucose and cellobiose in the reaction mixture have a detrimental effect on their activity. More precisely, these saccharification end products can bind to the active catalytic site of cellulases, and render them ineffective [54]. We assume that free glucose and cellobiose reduce the effective concentration of the enzymes available for saccharification, such that both inhibit EG and CBH, while BGL is only inhibited by glucose, as follows:

$$\left[EG\right]=[EG{]}_{0}-\omega_{EG}^{cbs}\frac{\left[EG{]}_{0}[cbs\right]}{\left[EG{]}_{0}+[CBH{]}_{0}+[cbs\right]}$$ (1)

$$-\omega_{EG}^{glc}\frac{\left[EG{]}_{0}[glc\right]}{\left[EG{]}_{0}+[CBH{]}_{0}+[BGL{]}_{0}+[glc\right]},$$

$$\left[CBH\right]=[CBH{]}_{0}-\omega_{CBH}^{cbs}\frac{\left[CBH{]}_{0}[cbs\right]}{\left[EG{]}_{0}+[CBH{]}_{0}+[cbs\right]}$$ (2)

$$-\omega_{CBH}^{glc}\frac{\left[CBH{]}_{0}[glc\right]}{\left[EG{]}_{0}+[CBH{]}_{0}+[BGL{]}_{0}+[glc\right]},$$

$$\left[BGL\right]=[BGL{]}_{0}-\omega_{BGL}^{glc}\frac{\left[BGL{]}_{0}[glc\right]}{\left[EG{]}_{0}+[CBH{]}_{0}+[BGL{]}_{0}+[glc\right]},$$ (3)

where, on the right-hand side of the equations, *[y]*_0_ denotes the concentration of the cellulases (i.e. *y* =  *EG*, *CBH*, or *BGL*) if inhibition would not take place, and [*x*] denotes the time varying concentration of the inhibitors (i.e. *x* =  *glc* for glucose and *cbs* for cellobiose). The parameter $\omega_{y}^{x}$ quantifies the inhibition strength of the inhibitor *x* on the enzyme *y*, and can vary between 0 and 1. These equations account for the competition of distinct inhibitors for the same active site of an enzyme, and conversely, for the sharing of common pools of inhibitor molecules by distinct enzymes.

In this study, we expand our model to additionally account for a higher level of detail relating to the crystalline and amorphous regions of the substrate. In the earlier version of the model [31], we assumed a simplistic homogeneous representation of cellulose and hemicellulose crystallinity, with the crystalline bonds being only located in the mid-length region of the microfibril, and the amorphous ones at the periphery. Nonetheless, during material preparation and pre-treatment (e.g. grinding to powder, and OrganoCat) the ordered structure of the crystalline cellulose and hemicellulose polymers may become disrupted, giving rise to random amorphous *defects*. The occurrence of such defects and disruption of crystallinity due to pre-treatments and mechanical stresses have also been reported in earlier studies [55–57]. These *defects* appear in the outer polymer chains of the microfibril, which are most exposed. In this expanded model, two independently adjustable parameters characterise the mean size $\left(\mu_{defect}\right)$ and the number (*N*_*defect*_) of such *defects*. $\mu_{defect}$ can vary in the range 0–0.5, denoting which fraction of the amorphous bonds in a polymer chain is embedded as *defects* in the crystalline region, instead of being located at the ends of the chain. *N*_*defect*_ varies in the range 0–1, quantifying which fraction of the outer polymer chains of a microfibril contains *defects*. Fig 1 shows a schematic representation of the entire microfibril, including amorphous *defects* located in the crystalline cellulose and hemicellulose regions. The distinct model parameters and their respective values are listed in the Sect 4.

### 2.6 Reminder of Gillespie algorithm

The enzymatic saccharification of the lignocellulose microfibril is simulated using a Gillespie algorithm, which is a common technique for implementing stochastic simulations. It mimics the stochastic nature of the system by considering a sequence of randomised events. Here, those can be the binding of enzymes to lignin, adsorption of CBH to a free polymer end, or reactions of enzymatic digestion. In our implementation of the Gillespie algorithm, for improved computational efficacy, we keep track of all and only the possible reactions, by accounting for the bond accessibility and steric hindrance. At each time step, both the reaction to take place and its duration are randomly selected, in a manner that reflects the reaction’s likelihood of occurrence, which can, for instance, depend on the substrate availability, the enzyme availability and the enzyme kinetics. The simulation lasts until the chosen amount of events is attained, or until all of the digestible substrate has been digested.

### 2.7 Fitting algorithm

In addition to our stochastic simulation model, we also developed a parameter fitting algorithm which enables us to search the parameter space to reproduce experimental saccharification time-course data. It proceeds through generations and sub-generations, with parameters randomly adjusted within specified ranges. The difference between experimental data and simulation curves (averaged over several runs) is recorded. If the difference decreases within a generation, the sub-generation with the lowest variance becomes the basis for the next generation. If the difference increases, the previous generation’s parameters are used again as a starting point for random adjustments. This mix of directed and random search ultimately converges to an optimal fit. The minimum difference across all generations defines the best fit for a particular set of experimental data. In this study, the model input parameters for the composition of the substrates are fixed to the experimentally measured values listed in Table 1. Then, in order to reproduce both the saccharification time-courses and the experimentally measured Crystallinity Indices, we fit the same kinetic parameters for all samples under a given pre-treatment condition (i.e. enzyme kinetic rates and end-product inhibition parameters), while the substrate related parameters are substrate specific (i.e. crystallinity fraction and digestibility ratio).

## 3 Results

### 3.1 Artificial cellulosic substrates

**Fig 2:**
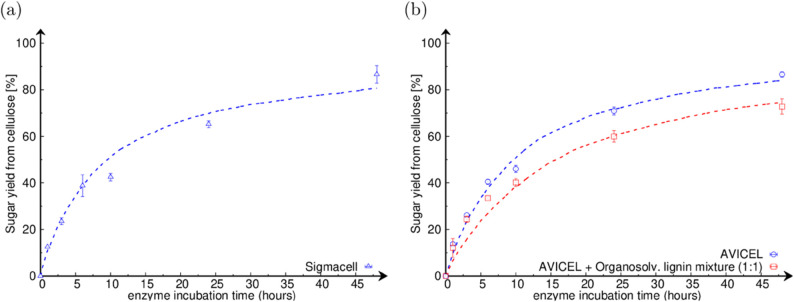
Enzymatic saccharification of cellulosic substrates. (a) Sigmacell; (b) in blue, AVICEL; in red, AVICEL and Organosolv lignin mixture (1:1). Experimental data are shown as points. Model simulation results are shown as dashed lines. The stochastic model fairly reproduces the saccharification dynamics in each case over the entire time-course.

Fig 2 displays the saccharification time-courses in three cases, i.e. for Sigmacell, AVICEL, and a (1:1) mixture of AVICEL with Organosolv lignin, with the experimental data being represented by points and the simulation results by dashed lines. We observe that the presence of Organosolv lignin in the reaction mixture decreases the overall sugar yield in comparison to pure AVICEL. In contrast to plant biomass, in which lignin is connected to cellulose, and hence physically blocks the access of cellulases to the cellulose polymers, the Organosolv lignin added to the cellulose in this case is not. To capture this, instead of a structured microfibril like shown in Fig 1, we consider polymers freely floating in the solution, where all their bonds can be easily accessed. In this configuration, lignin can still inhibit the cellulases by non-productively adsorbing on their surface, which we also represent in our model. Together panels (a) and (b) in Fig 2 show that the model is able to fairly reproduce the experimental time-courses, for each of the three cases considered.

As can be seen in Table 2, in the three cases considered in Fig 2, the Crystallinity Fraction of the model (averaged over the substrate) also fairly reproduces the Crystallinity Indices obtained by ssNMR. Since crystallinity in the model is quantified by two parameters: (i) the Crystallinity Fraction (noted CF) and (ii) the digestibility ratio (noted *r*_*c*,*a*_) (as described in Sect 2.5), this confirms that ssNMR quantifies the abundance of crystalline bonds in the total substrate, rather than their propensity to be digested. It is also interesting to note that while the Crystallinity Fraction of AVICEL (53%) is much higher than that of Sigmacell (22%), their sugar yields from cellulose at 48 hours are similar (86.6%). This non-trivial observation is well rationalised by the digestibility ratio of cellulose (cellu *r*_*c*,*a*_) determined by the model. For AVICEL, *r*_*c*,*a*_ = 0.60, while for Sigmacell, *r*_*c*,*a*_ = 0.20. It means that, the crystalline bonds are much harder to digest than the amorphous ones in Sigmacell as compared to AVICEL. This explains why a substrate with less crystalline bonds that are harder to digest (Sigmacell) releases the same amount of sugar like a substrate with more crystalline bonds that are easier to digest (AVICEL). This signifies that solely the measurement of the Crystallinity Index is not reflective of the amount of sugar being released from the substrate, which is instead influenced by a combination of the two factors: how much of the crystalline bonds are present (experimentally measured by Crystallinity Index and represented by the model parameter Crystallinity Fraction), and how hard they are to digest (represented by the model parameter digestibility ratio *r*_*c*,*a*_).

**Table 2:** Sugar yield from cellulose at 48 hours, experimentally measured Crystallinity Index (by ssNMR), and model parameters Crystallinity Fraction and digestibility ratio, from simulations that best fit saccharification time-courses in Fig 3.

Biomass source	Pre-treatment	Sugar yield from cellulose at 48 hrs [%]	Crystallinity Index from ssNMR [%]	Crystallinity Fraction [%]	$cellur_{c,a}$
Sigmacell	N.A	86.6±3.8	22.0±0.8	22.0	0.20
AVICEL	N.A	86.6±1.1	53.0±2.0	55.0	0.60
AVICEL and Organosolv Lignin	N.A	72.8±3.3	53.0±2.0	55.0	0.40
Beech wood	untreated	9.5±0.2	21.0±0.8	22.0	0.005
	NS+OCAT	50.8±0.4	43.0±1.6	45.0	0.04
	YS+OCAT	40.9±0.6	51.0±1.9	45.0	0.05
Miscanthus	untreated	11.5±0.6	29.0±0.8	31.0	0.05
	NS+OCAT	81.9±0.3	45.0±1.7	43.0	0.30
	YS+OCAT	47.0±4.4	30.0±1.1	33.0	0.001
Sida (Sida hermaphrodita)	untreated	12.4±0.5	23.0±0.9	22.0	0.05
	NS+OCAT	87.5±4.3	38.0±1.4	37.0	0.50
	YS+OCAT	40.4±1.4	34.0±1.3	36.0	0.001
Walnut shells	untreated	3.0±0.2	26.0±1.0	29.0	0.0001
	NS+OCAT	41.0±2.5	37.0±1.4	37.0	0.01
	YS+OCAT	22.6±1.0	47.0±1.7	45.0	0.0001

### 3.2 Plant biomass lignocellulosic substrates

In Table 2, we also consider various plant biomasses (i.e. beech wood, miscanthus, sida hermaphrodita (sida), and walnut shells), and conditions (i.e. no pre-treatment, OrganoCat pre-treatment without swelling (NS+OCAT), and OrganoCat pre-treatment with swelling (YS+OCAT)). We observe that the measured Crystallinity Indices from ssNMR for the untreated biomasses are always lower than that of the OrganoCat pre-treated samples. This is expected to be induced by the pre-treatment process, which removes a majority of the hemicelluloses from the biomass, and thereby amorphous polymers, leading to an increase in crystallinity of the overall substrate. For miscanthus and sida, the additional swelling step (YS+OCAT compared to NS+OCAT) swells crystalline cellulose and reduces the Crystallinity Indices. For beech wood and walnut shells, instead, during swelling amorphous cellulose is partially hydrolysed, which increases the overall Crystallinity Indices. To evaluate if any correlation can be drawn between the Crystallinity Index measured by ssNMR and the 48-hour sugar release (see Table 2), we rank them as follows:

**For untreated biomass:**

CI

beech wood(21.0±0.8)<sida(23.0±0.9)<walnut shells(26.0±1.0)<miscanthus (29.0 ± 0.8)

48-hour sugar release

sida(12.4±0.5)≈miscanthus(11.5±0.6)>beech wood(9.5±0.2)>walnut shells (3.0 ± 0.2)

**For NS+OCAT:**

CI

walnut shells(37.0±1.4)≈sida(38.0±1.4)<beech wood(43.0±1.6)≈miscanthus (45.0 ± 1.7)

48-hour sugar release

sida(87.5±4.3)>miscanthus(81.9±0.3)>beech wood(50.8±0.4)>walnut shells (41.0 ± 2.5)

**For YS+OCAT:**

CI

miscanthus(30.0±1.1)<sida(34.0±1.3)<walnut shells(47.0±1.7)<beech wood (51.0 ± 1.9)

48-hour sugar release

miscanthus(47.0±4.4)>beech wood(40.9±0.6)≈sida(40.4±1.4)>walnut shells (22.6 ± 1.0).

It is therefore apparent that, for the different biomasses considered, there is no correlation between the ranking of the measured Crystallinity Indices and their respective saccharification yields, which is another clue that the Crystallinity Index measured *via* ssNMR is insufficient to completely capture the biomass recalcitrance. Instead, one must also account for the digestibility ratio (*r*_*c*,*a*_) of the crystalline cellulose, that varies amongst biomasses.

**Fig 3:**
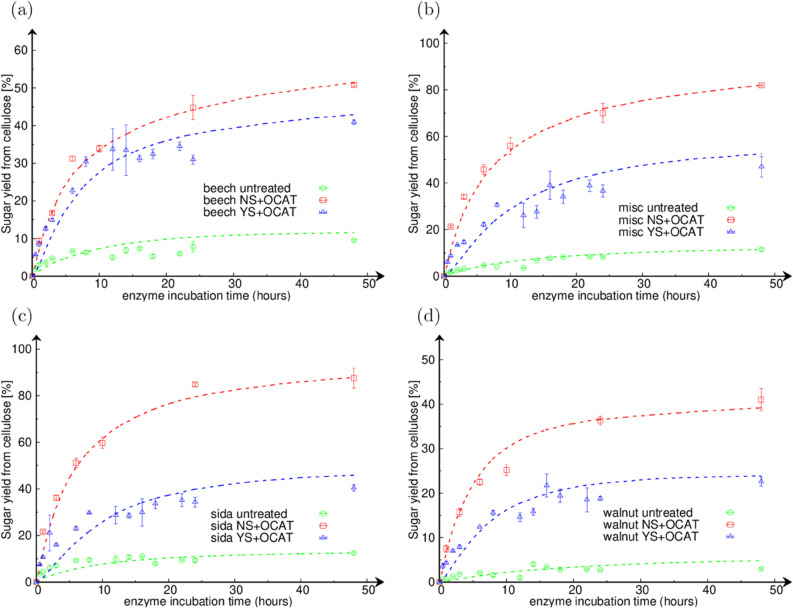
Enzymatic saccharification time-courses. The plots show the sugar release from cellulose *versus* the enzyme incubation time, for (a) beech (beech wood), (b) misc (miscanthus), (c) sida (Sida hermaphrodita), and (d) walnut (walnut shell) biomasses. Experimental data are shown as points and simulation results as dashed lines. Untreated biomass is in green, OCAT without swelling in red, and OCAT with swelling in blue.

Fig 3 displays the enzymatic saccharification time-courses of biomass from: (a) beech wood, (b) miscanthus, (c) sida, and (d) walnut shells, with experimental data being shown as points and simulation results as dashed lines. In each case, we observe that the untreated biomasses (in green) have the lowest sugar release; whereas the OrganoCat pre-treated ones without swelling (NS+OCAT, in red) have the highest, and the OrganoCat pre-treated ones with swelling (YS+OCAT, in blue) lie in between. In each case, the model fairly reproduces the experimental saccharification time-courses, while it takes into account the specific composition of the substrate (detailed in Table 1), and predicts a value for the model parameter Crystallinity Fraction that matches well the experimental Crystallinity Index, excepted for OrganoCat pre-treated beech wood with swelling (detailed in Table 2). In this case, the Crystallinity Fraction provided by the model is roughly 6% below the experimental Crystallinity Index. However, one could note that the impact of swelling on OrganoCat pre-treated beech wood is unique as compared to the other biomasses. Beech wood shows the lowest decrease in sugar release from cellulose when comparing NS+OCAT and YS+OCAT (i.e. ca. 9.9%), while this amounts to ca. 34.9% for miscanthus, ca. 47.1% for sida, and ca. 17.4% for walnut shells. The uniqueness of beech wood is also reflected in the fitted value of the cellulose digestibility ratio ($cellu\;r_{c,a}$), which unlike for other biomasses, is almost the same when comparing NS+OCAT and YS+OCAT conditions.

### 3.3 The digestibility ratio (*r*_*c*,*a*_)

In Table 2, considering all the samples investigated in this study, the $cellu\;r_{c,a}$ values indicate that the crystalline bonds in sida (NS+OCAT) are the easiest to enzymatically digest, while those in walnut shells (untreated and YS+OCAT) are the hardest. Interestingly, this correlates with highest sugar conversion at 48 hours for sida and lowest one for walnut shells. Additionally, all the samples showing more than 70% of sugar yield from cellulose at 48 hours have a $cellu\;r_{c,a}$ value in the range 0.2–0.6, while in all other cases $cellu\;r_{c,a}$ is at least one order of magnitude lower. It is therefore apparent that distinct samples (considering biomass and pre-treatment) have crystalline bonds with largely different digestibility ratios (*r*_*c*,*a*_), which strongly impacts, together with the Crystallinity Fraction, on the saccharification dynamics. Moreover, in the case of plant-sourced lignocellulosic biomasses, the digestibility ratios $cellu\;r_{c,a}$ follow the trend: NS+OCAT > untreated ≥ YS+OCAT, which suggests that $cellu\;r_{c,a}$ is a signature of the impact of the pre-treatment on the biophysical properties of the crystalline polymers.

It must be made explicit that the physical meaning of *r*_*c*,*a*_ is undoubtedly complex. Indeed, even though in the same chemical environment the same bonds need the same energy to be cleaved, there might be other factors such as the detailed spatial and structural arrangements of the molecules in and around the crystalline cellulose bonds which contribute to the biomass recalcitrance. For instance, the polysaccharide orientation, lignin composition and spatial arrangement of aromatic rings, lignin-carbohydrate-complexes, hemicellulosic decorations, or *kinks* in the linear arrangement of the cellulose chains may contribute to uneasiness for the enzymes to access and digest crystalline cellulose bonds. These features are neither accounted for in the model nor measured experimentally, and so, they might be captured, in a coarse-grained manner, by *r*_*c*,*a*_.

Noticeably, the model can also reproduce the experimental saccharification time-courses for the different biomasses and pre-treatment conditions using the same value of the digestibility ratio or the same value of the enzyme kinetics for all samples. However, then, the predicted values of the model parameter Crystallinity Fraction are very far from the experimental Crystallinity Indices. Thus, we instead have digestibility ratios that are specific for each biomass and pre-treatment, and enzyme kinetic rates that are common to all samples under a given pre-treatment condition. This confirms the impact of pre-treatments on the material properties and the consequent ability of an enzyme cocktail to digest it.

### 3.4 Further insights from the model

Apart from the crystallinity parameters (Crystallinity Fraction and digestibility ratio), other model parameters can have an impact on the saccharification dynamics [58]. For instance, below we illustrate the role of the end product inhibition and enzymes’ size, before summarising the overall phenomenology.

#### 3.4.1 Impact of cellulase inhibition.

**Fig 4:**
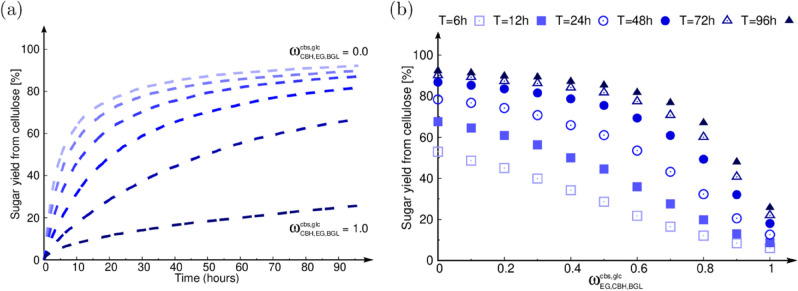
Simulated effect of end product inhibition on saccharification. (a) Saccharification time-courses with inhibition by both cellobiose and glucose. Each dashed line corresponds to a specific value for the inhibition factors, that are kept equal to one another. The inhibition factors are varied from $\omega_{y}^{x}=0$ to $\omega_{y}^{x}=1$ in steps of 0.2, where x=cbs,glc and y=EG,CBH,BGL. (b) Sugar released from cellulose *versus* $\omega_{y}^{x}$ at different time points along the simulated saccharification time-course. Each point type corresponds to a specific time.

Fig 4 shows the effect of the inhibition of cellulases by their end products cellobiose and glucose on the simulated saccharification dynamics. All the inhibition factors $\omega_{y}^{x}$ (see Sect 2.5) are kept equal to each other, and simultaneously varied. All the other parameters are kept fixed to the best-fitted values for the case of miscanthus OrganoCat pre-treated without swelling (which we arbitrarily picked). In Fig 4a, as one might intuitively expect, we observe that, as the value of the inhibition factor increases, the sugar yield from cellulose decreases. In Fig 4b, we study the effect of the end-product inhibition factors $\omega_{y}^{x}$ on the sugar release at different points of the time-course. At earlier times, an increase in the inhibition causes a pseudo-linear decrease in the sugar release. At later times, two regimes are observed: first a very slow decrease of the sugar release with increasing $\omega_{y}^{x}$, second, a sharp decrease of the sugar release with increasing $\omega_{y}^{x}$. Additionally, when comparing the sugar yield from cellulose at $\omega_{y}^{x}=0$ and $\omega_{y}^{x}=1$, we clearly observe that this difference is stronger for late time points than for early time points, which highlights the cumulative impact of inhibition throughout the saccharification time-course. We also tested the cases where the only inhibitor was either cellobiose or glucose (data not shown). We observed that inhibition by only cellobiose has a negligible effect on the saccharification dynamics, since it does not accumulate and is instead readily digested into glucose by BGL. A very similar, but weaker, profile like in Fig 4 is observed when only glucose inhibits, hence, we deduce that inhibition by cellobiose reinforces that by glucose.

#### 3.4.2 Effect of enzymes’ size.

**Fig 5:**
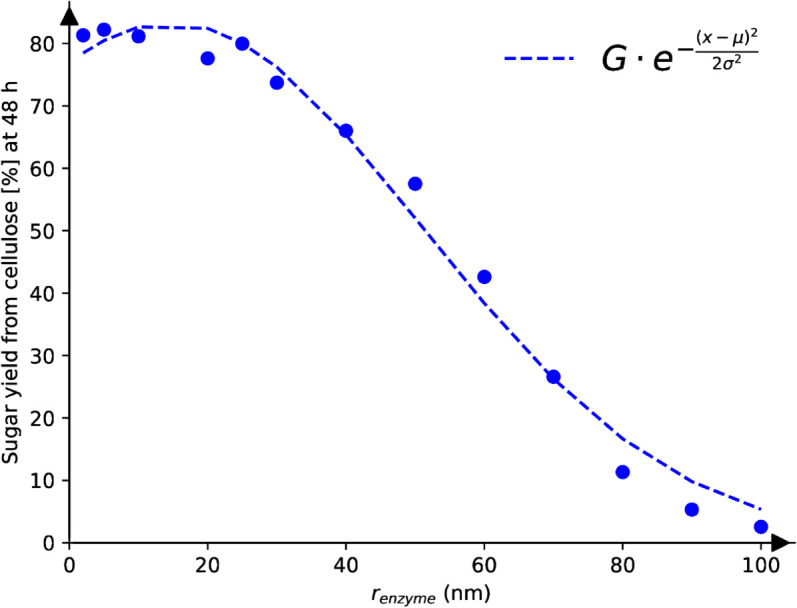
Simulated effect of the enzymes’ size on the saccharification dynamics. Sugar yield from cellulose at 48 hours decreases with increasing enzymes’ size, following a Gaussian trend (G=83.32±1.65,μ=14.62±2.25,σ=36.43±2.06).

Fig 5 shows the effect of varying enzymes’ size on the simulated saccharification dynamics. All the other model parameters are fixed to the best-fitted values for miscanthus OrganoCat pre-treated without swelling (which we arbitrarily picked). The enzymes are approximated as hard spheres, with a radius of *r*_*enzyme*_, such that they interact among themselves and with the substrate through excluded-volume interactions. Due to their intrinsic volume, both cellulases and hemicellulases may have limited access to their respective substrate because it is being shielded by surrounding non-substrate polymers. Furthermore, processive enzymes (i.e. CBH) that remain attached to the microfibril for a while, prevent other enzymes from approaching and digesting bonds. As a consequence, we observe that steric hindrance increases with the enzyme radius, which directly slows down the saccharification process. The sugar yield from cellulose at 48 hours can be fitted with the right side of a Gaussian distribution, using the parameters stated in the caption of Fig 5.

#### 3.4.3 Overall phenomenology.

**Fig 6:**
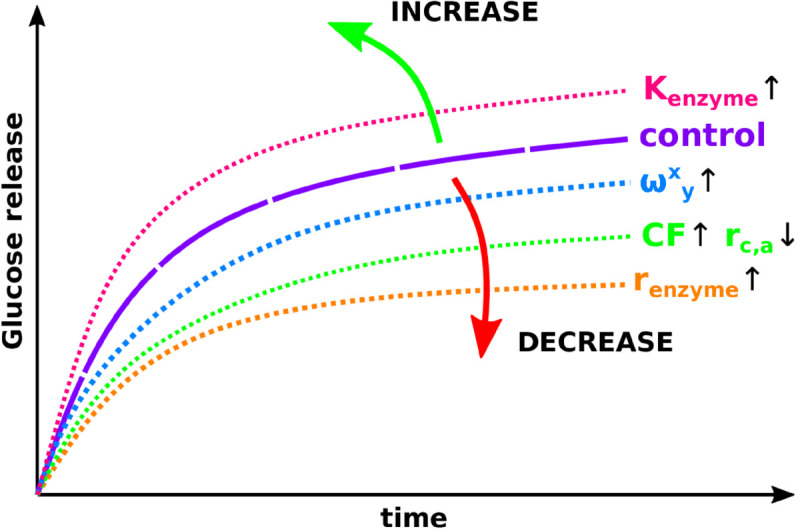
Representative plot showing the impact of several model parameters on the simulated saccharification time-courses, in comparison to a control case (purple line), considering a generic lignocellulose substrate. The depicted parameters are: the enzyme reaction rates (noted *K*_*enzyme*_, pink line), the end product inhibition parameters (noted $\omega_{y}^{x}$, blue line), the enzymes’ size (noted *r*_*enzyme*_, orange line), and the substrate crystallinity parameters (noted *CF* and *r*_*c*,*a*_, green line).

Fig 6 illustrates how the various model parameters affect the saccharification time-courses for a generic lignocellulose substrate. We assume, for each line, the variation of only the marked parameter, with respect to the control, while all the other parameters, including the substrate composition, are considered to be constant. The saccharification yield increases with the increase in the enzyme reaction rates (*K*_*enzyme*_), or with the decrease in the digestibility ratio (*r*_*c*,*a*_). Conversely, the saccharification yield decreases with the increase of the end product inhibition ($\omega_{y}^{x}$), or with that of the Crystallinity Fraction (*CF*), or with that of the enzymes’ radius (*r*_*enzyme*_). These generic considerations are presented for a control sample, whose substrate composition is kept fixed. In order to fully rationalise experimental saccharification time-courses (such as in Fig 3), one should be reminded that the variation in substrate composition can also play a significant role on its recalcitrance, in particular when considering biomass from different sources.

## 4 Discussion

In this study, we use a two-fold computational and experimental approach to investigate the enzymatic saccharification of not only purely cellulosic substrates (i.e. AVICEL and Sigmacell) but also lignocellulosic biomasses from four different plant sources (i.e. beech wood, miscanthus, sida, and walnut shells), that we additionally examine under three distinct conditions (i.e. untreated, OrganoCat pre-treated without swelling, and OrganoCat pre-treated with swelling). We employ an upgraded version of our earlier published stochastic biophysical model [31] to reproduce the experimental saccharification time-courses of the fifteen samples while taking into account their specific composition. Our best-fits provide values for the model parameter Crystallinity Fraction that match well the Crystallinity Indices experimentally measured by ssNMR. The enzymatic saccharification of AVICEL in solution with Organosolv lignin highlights the inhibitory effect of lignin on saccharification due to its adsorption on the enzymes, even though it is not physically connected to the cellulosic substrate in that case. For the four plant-sourced biomasses, the pre-treatment consistently impacts on the sugar release. Compared to untreated biomasses, OrganoCat pre-treatment brings about a many-fold increase in the saccharification yield. Untreated biomasses have the lowest yield, OrganoCat pre-treated with swelling the intermediate one, and OrganoCat pre-treated without swelling the highest one.

The Crystallinity Index of distinct plant-sourced biomasses can be differently affected by swelling, that can either increase or decrease it. Additionally, with both plant-sourced biomasses and purely cellulosic substrates (i.e. AVICEL and Sigmacell), we highlight that the Crystallinity Index is not correlated to the sugar release that is instead determined by a combination of the Crystallinity Fraction (that is the model parameter for the experimentally determined Crystallinity Index) and the digestibility ratio. This clearly suggests that the Crystallinity Index measured by ssNMR is important but insufficient to predict the saccharification yield of a given biomass. Therefore, the digestibility ratio appears as an essential parameter to rationalise the saccharification dynamics, and while it can be different for distinct biomasses, it emerges as a footprint of the impact of pre-treatments on the material. Yet, its exact physical meaning is undoubtedly complex, and can be considered as a coarse-grained representation of various spatial and structural features of the material that explain why crystalline bonds are more or less easily digested in distinct biomasses. Other model parameters also impact on the saccharification dynamics, in particular the end product inhibition and the enzyme size. Although resulting from very distinct mechanisms, based on the simulations, we show that either an increase in end product inhibition or enzyme size consistently reduces the saccharification yield in a non-trivial fashion.

Overall, our study illustrates the benefit of modelling to rationalise complex experimental data related to the dynamics of lignocellulose biomass saccharification. Importantly, it also stresses the need for further experimental investigations towards deciphering the fine structural properties of the material that explain why the digestibility of crystalline regions vary that much across biomasses and pre-treatment conditions. In this perspective, complementary approaches could be based on lower scale modelling, such as atomistic-level Molecular Dynamics or Density Field Theory.

## Appendix

### Glossary

**List of symbols**



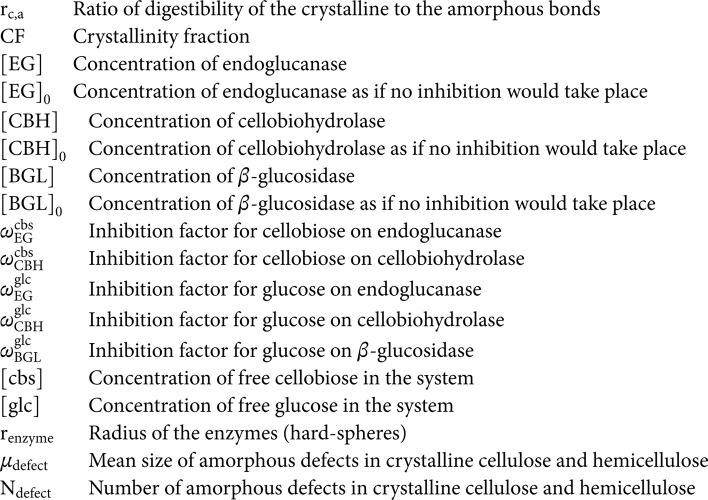



**List of abbreviations**

DP: degree of polymerisation, EG: endoglucanase, CBH: cellobiohydrolase, BGL: *β*-glucosidase, HC: hemicellulase, MLG: mixed-linkage glucan, DFT: density functional theory, MD: molecular dynamics, MPS: matrix polysaccharide, AIR: alcohol insoluble residue, ssNMR: solid-state nuclear magnetic resonance, OCAT: OrganoCat, OCAT+NS: OrganoCat No-Swelling, OCAT+YS: OrganoCat Yes-swelling.

### Input parameters and files of the stochastic biophysical model

The model source code available in the GitLab repository is organised such that input parameters are read as text files, which are in the folder ‘Params’. These files are named ‘kinetic_parameters.txt’ and ‘initial configuration_parameters_*.txt’. The parameters are listed as comments at the bottom of the respective files for ease of the user. We also list them below.

**Parameters listed in the input file ‘kinetic_parameters.txt’:**

➯ **EG *K***_***cat***_
   parameter #1
   Endoglucanase catalytic constant.
   Units: 1/s. Typical Range: 0.001-1000.
➯ **EG *K***_***m***_
   parameter #2
   Endoglucanase Michaelis-Menten constant.
   Units: mM. Typical Range: 0.001-1000.
➯ **CBH processive digestion rate**
   parameter #3
   Reaction rate of cellobiohydrolase processive action.
   Units: reactions/hour. Typical Range: 10-10,000.
➯ **BGL *K***_***cat***_
   parameter #4
   *β*-glucosidase catalytic constant.
   Units: 1/s. Typical Range: 0.001-1000.
➯ **BGL *K***_***m***_
   parameter #5
   *β*-glucosidase Michaelis-Menten constant.
   Units: mM. Typical Range: 0.001-1000.
➯ **XYL *K***_***cat***_
   parameter #6
   Xylanase catalytic constant.
   Units: 1/s. Typical Range: 0.001-1000.
➯ **XYL *K***_***m***_
   parameter #7
   Xylanase Michaelis-Menten constant.
   Units: mM. Typical Range: 0.001-1000.
➯ **CBH *K***_***cat***_ (attachment reaction)
   parameter #8
   Cellobiohydrolase catalytic constant.
   Units: 1/s. Typical Range: 0.001-1000.
➯ **CBH *K***_***m***_ (attachment reaction)
   parameter #9
   Cellobiohydrolase Michaelis-Menten constant.
   Units: mM. Typical Range: 0.001-1000.
➯ **inhibition binding affinity of cellobiose to EG**
   parameter #10
   $\omega_{EG}^{cbs}$
   Units: none. Range: 0-1.
➯ **inhibition binding affinity of cellobiose to CBH**
   parameter #11
   $\omega_{CBH}^{cbs}$
   Units: none. Range: 0-1.
➯ **inhibition binding affinity of glucose to EG**
   parameter #12
   $\omega_{EG}^{glc}$
   Units: none. Range: 0-1.
➯ **inhibition binding affinity of glucose to CBH**
   parameter #13
   $\omega_{CBH}^{glc}$
   Units: none. Range: 0-1.
➯ **inhibition binding affinity of glucose to BGL**
   parameter #14
   $\omega_{BGL}^{glc}$
   Units: none. Range: 0-1.
➯ **Enzyme size: radius**
   parameter #15
   *r*_*enzyme*_
   Units: nm. Value used: 2.5 (based on formula 2.2 in [59] and enzyme molecular masses from BRENDA.)

Parameters listed in the input file ‘initial_configuration_parameters_*.txt’:

➯ **mode_code**
   parameter #1
   Determines the shape of the microfibril and the number of cellulose polymers present.
   Range: 1-5 (integers only). Mode code = 1 or 2, 24 polymers; Mode code = 3 or 4 (used here), 18 polymers; Mode code = 5, 36 polymers.
➯ **pct_EG**
   parameter #2
   Relative fraction of endoglucanase in the enzyme cocktail.
   Units: none. Value used: 0.135 (based on the most efficient enzyme cocktail design from literature [60,61] and calculations in [31]). Range: 0-1.
➯ **pct_CBH**
   parameter #3
   Relative fraction of cellobiohydrolase in the enzyme cocktail.
   Units: none. Range: 0-1. Value used: 0.353 (based on the most efficient enzyme cocktail design from literature [60,61] and calculations in [31]).
➯ **pct_BGL**
   parameter #4
   Relative fraction of *β*-glucosidase in the enzyme cocktail.
   Units: none. Range: 0-1. Value used: 0.100 (based on the most efficient enzyme cocktail design from literature [60,61] and calculations in [31]).
➯ **pct_XYL**
   parameter #5
   Relative fraction of xylanase in the enzyme cocktail.
   Units: none. Range: 0-1. Value used: 0.412 (based on the most efficient enzyme cocktail design from literature [60,61] and calculations in [31]).
➯ **total_enz_molecules**
   parameter #6
   Total number of enzyme molecules involved in the digestion of the single simulated microfibril.
   Units: none. Value used: 50.
➯ **length_fibril**
   parameter #7
   Length of the simulated microfibril in terms of *β*-1-4 bonds in the cellulose chain.
   Units: none. Value used: 200.
➯ **boolean_Xyl_or_MLG**
   parameter #8
   It determines whether the hemicellulose contains either xylose(1) or mixed-linkage glucans(0).
   Units: none. Value used: 1.
➯ **pct_xyl**
   parameter #9
   Percentage of xylose in the hemicellulose.
   Value: 1 (Currently unused, kept as a placeholder for future upgrades to accommodate multiple significant hemicellulose types at the same time).
➯ **pct_cellu**
   parameter #10
   Relative fraction of cellulose in the substrate composition.
   Unit: none. Range: 0.01-1 (value as user input from Table 1).
➯ **pct_hemi**
   parameter #11
   Relative fraction of hemicellulose in the substrate composition.
   Unit: none. Range: 0.01-1 (value as user input from Table 1).
➯ **pct_lign**
   parameter #12
   Relative fraction of lignin in the substrate composition.
   Unit: none. Range: 0.01-1 (value as user input from Table 1).
➯ **pct_acetyl_hemi**
   parameter #13
   Currently unused. Value: 0
➯ **pct_crystalline_cellu**
   parameter #14
   CF cellulose.
   Units: none. Range: 0-1. (Fitted uniquely per sample to reproduce saccharification timecourses and CI of total biomass)
➯ **pct_crystalline_hemi**
   parameter #15
   CF hemicellulose.
   Units: none. Range: 0-1. (Fitted uniquely per sample to reproduce saccharification timecourses and CI of total biomass)
➯ **Mean_defect_size**
   parameter #16
   $\mu_{defect}$
   Units: none. Range: 0-0.5.
➯ **Nbr_of_defects**
   parameter #17
   $N_{defect}$
   Units: none. Range: 0-1.
➯ **r_monomer**
   parameter #18
   Radius of a single monomer of cellulose and hemicellulose.
   Units: nm. Value fixed: 0.6.
➯ **Lignin_adhesion_rate**
   parameter #19
   The number of monolignols that undergo non-productive adsorption on a single enzyme molecule. A smaller number indicates higher non-productive adsorption.
   Units: none. Typical Range: 100-350.
➯ **digestibility_ratio cellu**
   parameter #20
   *r*_*c*,*a*_ cellulose.
   Units: none. Range: 0.00001-1. (Fitted uniquely per sample to reproduce saccharification timecourses and CI of total biomass)
➯ **digestibility_ratio hemi**
   parameter #21
   *r*_*c*,*a*_ hemicellulose.
   Units: none. Range: 0.00001-1. (Fitted uniquely per sample to reproduce saccharification timecourses and CI of total biomass)
